# Molecular prevalence of *Chlamydia* spp. in wild birds from Qinghai Lake, China

**DOI:** 10.1186/s13620-025-00314-2

**Published:** 2025-10-30

**Authors:** Xiaomin Wu, Fan Lei, Yaqian Niu, Jiali Yu, Chao Chen, Te Ba, Lin Liang

**Affiliations:** 1 State Key Laboratory of Plateau Ecology and Agriculture, Qinghai University, 810016 Xining, China; 2 Department of Animal Medicine, College of Agriculture and Animal Husbandry, Qinghai University, Xining, 810016 China; 3 Hainan Animal Disease Prevention and Control Center, Gonghe, Qinghai province 813000 China

**Keywords:** Molecular, *Chlamydia* spp, Wild birds

## Abstract

*Chlamydia* spp. are a group of gram-negative, obligate intracellular bacteria that represent significant pathogens causing chlamydiosis in both animals and humans. Avian chlamydiosis (AC), primarily caused by *Chlamydia psittaci*, *C. avium*, *C. gallinacea*, and *C. ibidis*, has been documented in over 460 avian species. Qinghai Lake, China’s largest saltwater lake and a critical overwintering site for migratory birds, served as the study area to investigate *Chlamydia* prevalence in wild birds. Fecal samples from 125 birds revealed an overall *Chlamydia* spp. infection rate of 28.8% (36/125), with three species identified: *C. abortus* (55.6%, 20/36), *C. avium* (44.4%, 16/36), and *C. psittaci* (13.9%, 5/36). Phylogenetic analysis through amplification of the 16 S rRNA (5 samples), IGS-23 S rRNA (6 samples), and *ompA* (5 samples) genes revealed that all sequences obtained in this study were assigned to the *Chlamydiaceae* family. The *ompA* sequence of *C. abortus* obtained in this study clustered closely with the reference strain GN6 (CP021996.1) isolated from aborted yak fetuses (*Bos grunnien*s), a bovid species typically domesticated and endemic to the Qinghai-Tibet Plateau, showing 100% sequence identity. In contrast, *C. avium ompA* sequences shared 87.40% identity with the reference strain 10DC88. The identical genomic profile of *C. abortus* between wild birds and domesticated yaks suggests potential cross-species transmission in the Qinghai-Tibetan Plateau, where overlapping habitats of wildlife and livestock may facilitate pathogen exchange. These findings underscore the zoonotic risks posed by *Chlamydia* species circulating in this ecologically critical region, with implications for the health of local livestock (yaks, Tibetan sheep), human populations, and migratory bird conservation.

**Supplementary Information:**

The online version contains supplementary material available at 10.1186/s13620-025-00314-2.

## Background

*Chlamydia* spp. are a group of gram-negative, obligate intracellular bacteria that causes chlamydiosis in animals and humans. Among these, *C. psittaci*, *C. avium*, *C. gallinacea* and *C. ibidis* are recognized as the causative agents of avian chlamydiosis (AC). *C. psittaci* is classified into 9 classical genotypes (A-F, E/B, WC, and M56) based on restriction fragment length polymorphism (RFLP) analysis of the *ompA* gene. Subsequent studies analyzing *ompA* sequences from wild birds have identified atypical genotypes, including 1 V, 6 N, Mat116, R54, YP84, and CPX0308 [1]. *C. psittaci* exhibits broad host specificity, infecting over 460 avian species [2]. Infected birds commonly present with respiratory distress, anorexia, emaciation, diarrhea, and, in severe cases, fatal outcomes. Additionally, *C. psittaci* poses significant zoonotic risks, threatening public health globally [3]. Human infections, often reported among poultry workers exposed to aerosols from contaminated feces or respiratory secretions, typically manifest as fever, cough, headache, and severe respiratory illnesses [4].

The global prevalence of *C. psittaci* in avian populations is notably high. Epidemiological surveys across 26 countries on five continents report an average infection rate of 19.5% [5]. Documented cases span diverse regions, including Egypt, Argentina, the Netherlands, and New Zealand, where varying infection rates have been observed in domestic birds [4, 6–8]. Notably, *C. psittaci* has also been detected in non-avian hosts such as horses and cats [9, 10]. In China, multiple studies highlight its widespread prevalence: a 31.09% seropositivity rate in northwest-region pigeons [11], a 26.2% positivity rate (602/2300) in poultry across 24 provinces [12], a 22.9% infection rate in cattle from 11 provinces [13], and a 34.2% detection rate in Taiwanese waterfowl farms [14]. Furthermore, *C. psittaci* was identified in 20 of 99 fecal samples from crested ibis [15], underscoring its pervasive presence in Chinese livestock and poultry.

Beyond *C. psittaci*, emerging species such as *C. buteonis*, *C. ibidis*, *C. gallinacea*, and *C. avium* have been linked to avian conjunctivitis and respiratory ailments. *C. avium*, primarily reported in European pigeons and parrots, was detected in 36.6% and 20.0% of pigeon droppings from Utrecht and Haarlem, respectively [8]. In China, surveys of chickens, ducks, geese, and pigeons revealed *C. gallinacea*, *C. psittaci*, *C. suis*, *C. muridarum*, and *C. pecorum* as predominant species, with *C. gallinacea* and *C. psittaci* occurring across all four avian hosts. Notably, no *C. avium* strains were identified in these studies [12].

Qinghai Lake, the largest saltwater lake in China, supports rich biodiversity and serves as a critical habitat for migratory waterbirds, including black-necked cranes (*Grus nigricollis*), pallas’s gull (*Larus ichthyaetus*), bar-headed geese (*Anser indicus*), brown-headed gulls (*Chroicocephalus brunnicephalus*), common cormorants (*Phalacrocorax carbo*), and whooper swans (*Cygnus cygnus*). Annually, approximately 4,000 whooper swans overwinter in this region. Despite its ecological significance, no data are available on *Chlamydia* prevalence in Qinghai Lake’s wild bird populations. This study aims to characterize *Chlamydia* species diversity through fecal sampling in the area. By enhancing diagnostic capabilities and informing disease control strategies, the findings will provide a scientific foundation for mitigating *Chlamydia*-related zoonotic risks, thereby offering significant public health and ecological applications.

## Materials and methods

### Study site and sample collection

**Fig. 1:**
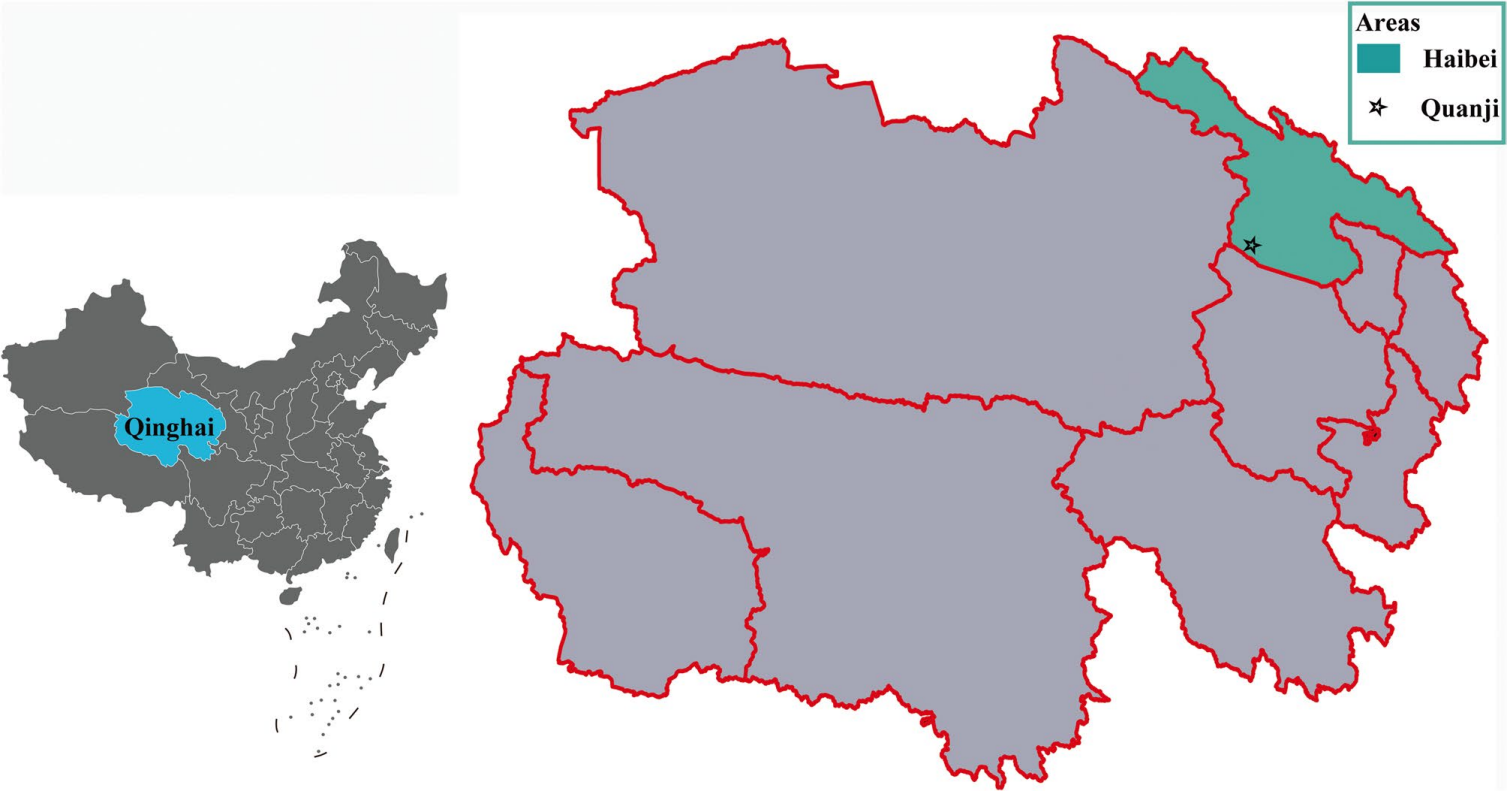
A map of China showing Qinghai Province and the geographical regions where wild bird feces were collected

Sampling was conducted at Qinghai Lake Nature Reserve, located in Quanji Township, Gangcha County, Haibei Tibetan Autonomous Prefecture, Qinghai Province, situated on the northwestern shore of Qinghai Lake. It forms an integral part of the Qinghai Lake National Nature Reserve. The coordinates 99°58′E and 37°23′N (Fig. 1), with an elevation of approximately 3,200 m above sea level. The wetlands surrounding Quanji Township serve as critical breeding and stopover grounds for rare waterbirds such as the bar-headed goose, whooper swan, and black-necked crane.

Sampling was conducted at three distinct intervals: on October 1–3, October 12–14, and October 23–25 in 2023. A total of 125 fecal samples were collected from wild birds. The fecal samples encompassed 3 different avian species, including bar-headed goose, pallas’s gull, and brown-headed gull. Fecal samples were collected using sterile cotton-tipped swabs that were immediately placed into 2 mL sterile centrifuge tube. Species identification of the source birds was determined by a combination of factors: (1) sampling was conducted in specific areas habitually used and dominated by one of the three target species (bar-headed goose, pallas’s gull, or brown-headed gull), and (2) immediate collection of fresh feces after the birds were observed to leave the spot, with identification confirmed by the extensive field experience of the reserve staff in recognizing avian droppings. While this is a standard non-invasive method for field ecology studies, we acknowledge its potential limitations. Ethical approval was not required for this observational study, as samples were non-invasively collected by reserve staff during routine sanitation procedures. To preserve *Chlamydia* viability and diversity, fresh fecal materials were prioritized during collection. All samples were immediately frozen at − 80 °C until processing.

### DNA extraction

Fecal swabs were immersed in 1 mL phosphate-buffered saline (PBS), vortexed for 10 min, and thoroughly squeezed to remove residual liquid. Genomic DNA was extracted using the TIANamp Genomic DNA Kit (TIANGEN, China), eluted in 120 µL of elution buffer, and stored at − 20 °C until use.

### Real-time PCR assays to identify *Chlamydiaceae* species and DNA sequencing

All DNA samples (*n* = 125) underwent preliminary screening with a *Chlamydiaceae*-specific 23 S rRNA-targeting qPCR assay as described by Ehricht et al. [16]. *Chlamydiaceae*-positive samples were subsequently tested in duplicate using species-specific qPCR protocols for *C. pecorum* [17], *C. abortus* [17], *C. psittaci* [17], *C.avium* [18], *C. gallinacea* [18], and *C. pneumoniae* [19]. Following Mitura et al.‘s criteria [20], a cycle threshold (Ct) cut-off value of 38.0 was applied, corresponding to the assay’s lower detection limit. Positive DNA samples were further confirmed by amplification and sequencing of specific fragments of *ompA*, 16 S rRNA, and 16 S rRNA-23 S rRNA intergenic spacer together with 23 S rRNA domain I (IGS-23 S rRNA) as described previously [21–23]. PCR products were sequenced (Tsingke Biotechnology Co., Ltd., Xi’an, China). The obtained sequences were deposited in the GenBank database with the following accession numbers.

### Phylogenetic analysis

Five 16 S rRNA sequences, six IGS-23 S rRNA sequences, and five *ompA* sequences (GenBank: PQ248390–PQ248396, PQ276123–PQ276128, PQ303604–PQ303608) were analyzed using MEGA 7.0. Sequence homology was verified via BLASTn against NCBI databases, followed by multiple alignment with reference *Chlamydia* strains. Phylogenetic trees for 16 S rRNA (1,400 bp), IGS-23 S rRNA (1,000 bp), and *ompA* (1,050 bp) were reconstructed using the neighbor-joining method with 1,000 bootstrap replicates under the Maximum Composite Likelihood model.

## Results

**Fig. 2:**
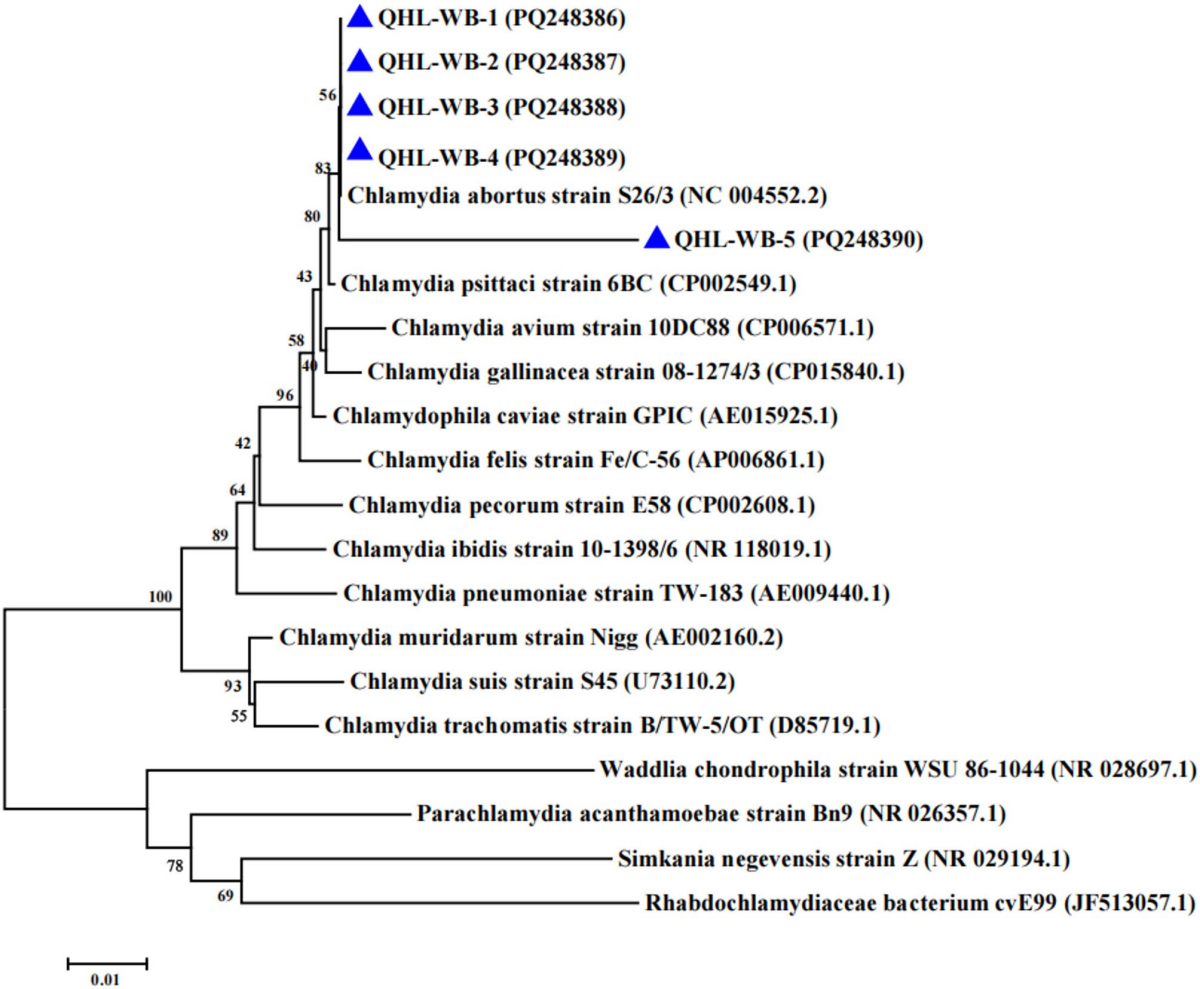
Phylogenetic tree based on the 16S rRNA gene fragment (about 1400 bp) of *Chlamydia*. The phylogenetic tree includes representative sequences of established *Chlamydiaceae* species sequences as well as sequences discovered in this study (GenBank accession number was marked by blue triangle) were included *Parachlamydia acanthamoebae* strain Bn9, *Rhabdochlamydiaceae* bacterium cvE99, *Simkania negevensis* strain Z and *Waddlia chondrophila* strain WSU 86–1044 were used as an outgroup. Based on these alignments, phylogenetic trees were constructed by the neighbour-joining method using the Maximum Composite Likelihood model with MEGA 7.0.

**Fig. 3:**
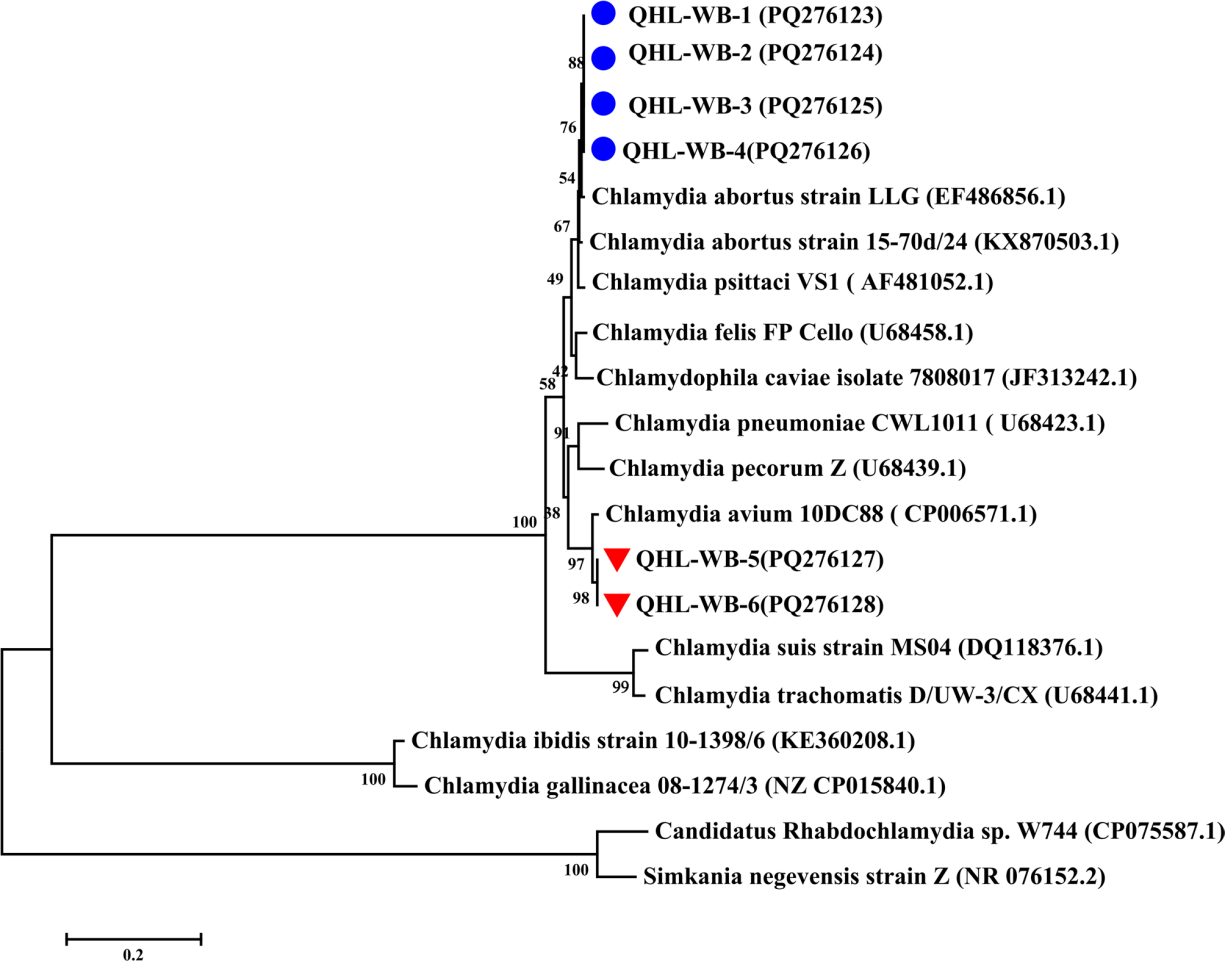
Phylogenetic tree based on the 16S-23S intergenic spacer and full length of 23S rRNA domain I fragment (about 910 bp) of *Chlamydia*. The phylogenetic tree includes representative sequences of established *Chlamydiaceae* species sequences as well as sequences discovered in this study (GenBank accession number was marked by blue circles and red triangles) were included *Candidatus Rhabdochlamydia* sp. W744 and *Simkania negevensis* strain Z were used as an outgroup. Based on these alignments, phylogenetic trees were constructed by the neighbour-joining method using the Maximum Composite Likelihood model with MEGA 7.0.

**Fig. 4:**
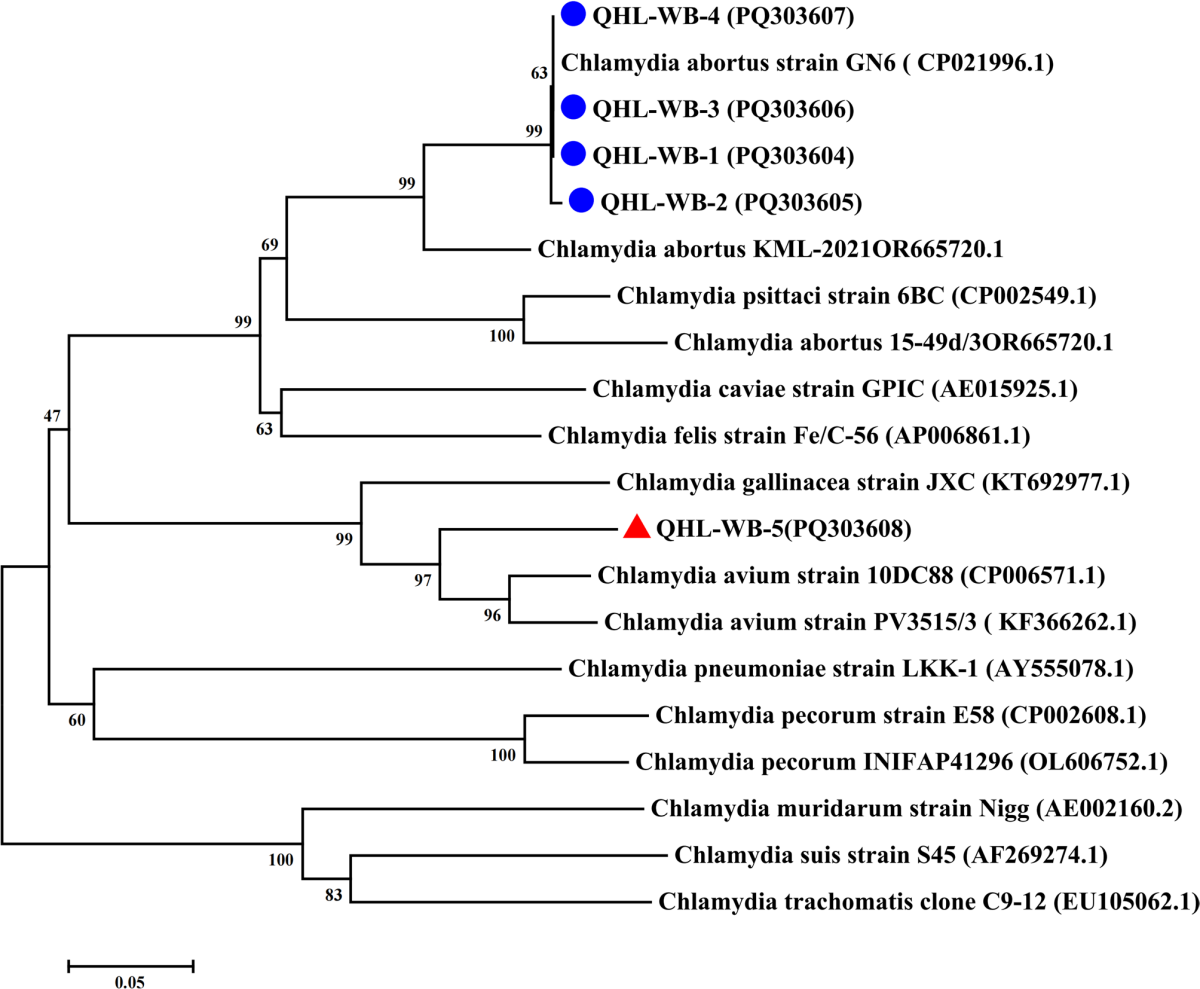
Phylogenetic tree based on the *ompA* gene fragment (about 950 bp) of *Chlamydia*. The phylogenetic tree includes representative sequences of established *Chlamydiaceae* species sequences as well as sequences discovered in this study (GenBank accession number was marked by blue circles and red triangles). Based on these alignments, phylogenetic trees were constructed by the neighbour-joining method using the Maximum Composite Likelihood model with MEGA 7.0.

By screening 125 wild bird fecal samples, *Chlamydia* spp. were detected in a total of 36 fecal samples with an overall prevalence of 28.8% (36/125). Three species were identified: *C. abortus* (55.6%, 20/36), *C. avium* (44.4%, 16/36), and *C. psittaci* (13.9%, 5/36), while *C. pecorum*, *C. gallinacea*, and *C. pneumoniae* were undetected (Table 1). The distribution of these pathogens across the thress bird species is detailed in Table 2. As shown, *C.abortus* was the most frequently detected species across all host birds. To further confirm the presence of the *Chlamydia* spp., 16 S rRNA (5 samples), IGS-23 S rRNA (6 samples), and *ompA* (5 samples) gene amplicons were successfully amplified and sequenced from high-concentration positive samples. Comparative phylogenetic analysis demonstrated that all sequences were assigned to the *Chlamydiaceae* family. *C. abortus* and *C. avium* identified in this study were all closely related to their respective reference sequences available in GenBank and altogether showed a similar topology (Figs. 2, 3 and 4). The *ompA* sequence of *C. abortus* obtained in this study clustered closely with the reference strain GN6 (CP021996.1) isolated from aborted yak fetuses, showing 100% sequence identity, while *C. avium*showed high genetic diversity, and sharing 87.40% identity with *C. avium* reference strain 10DC88 (Figure S1).

**Table 1:** Detection of *Chlamydia* in bird feces in Qinghai lake area

District	Sample type	Chlamydia positive sample/sample number	Number of positive samples for Chlamydia species/Number of positive samples for Chlamydia family		
			Chlamydia abortus	Chlamydia avium	Chlamydia psittaci
Qinghai Lake	feces	36/128	20/36	16/36	5/36

**Table 2:** Prevalence of *Chlamydia* species in wild bird species from Qinghai lake

Bird Species	Chlamydia positive samples/no. of samples	No of Chlamydia spp positive samples		
		C.abortus	C.avium	C.psittaci
Bar-headed goose (*Anser indicus*)	15/52	10	7	2
Pallas’s gull (*Larus ichthyaetus*)	12/38	5	5	2
Brown-headed gull (*Chroicocephalus brunnicephalus*)	9/35	5	4	1
Total	36/125	20	16	5

Co-infections with multiple *Chlamydia* species were detected in some samples, therefore the sum of the individual species counts may exceed the total number of positive samples

## Discussion

This study investigating *Chlamydiaceae* prevalence in wild birds of Qinghai Lake, China, revealed an overall infection rate of 28.8% (36/125). Three species were identified: *C. abortus* (55.6%, 20/36), *C. avium* (44.4%, 16/36), and *C. psittaci* (13.9%, 5/36). Notably, this constitutes the inaugural documentation of *Chlamydiaceae* in Qinghai Lake’s avian populations and highlights *C. abortus* as a novel avian pathogen in China. Our findings align with domestic poultry surveillance data from China (26.2%, 602/2,300) [12], yet exceed rates reported in Brazilian backyard chickens (25.2%, 60/238) [24], Swiss turkeys (0.7%, 7/1008) [25], and endangered Crested Ibis populations (20.2%, 20/99) [15]. Discrepancies may arise from variations in host susceptibility, sampling strategies (e.g., fecal vs. cloacal swabs), and diagnostic methodologies (qPCR vs. ELISA).

Although AC caused by *C. psittaci* and emerging species like *C. avium* is typically subclinical but occasionally fatal [26–28]. While our data revealed a relatively low *C. psittaci* prevalence (13.9%) compared to regional poultry studies. Specifically, prevalence rates in Changsha poultry (27.5%) [29], Hainan domestic geese (25.6%) [30], Lanzhou pigeons (31.09%), Weifang and Beijing cities parrots (35.37%) [11, 31], exceeded our observations, potentially due to higher host density. Conversely, our rates surpassed those reported in Swedish garden birds (2.2%), Brazil backyard chickens (0.0%), Sichuan and Shandong birds (3.13%), and Hong Kong pet birds (0.97%) [15, 24, 32, 33]. This difference may be related to the ecological niche differences between wild and captive populations. These comparative analyses underscore the importance of host species ecology in chlamydial epidemiology.

In this study, our data showed that *C. abortus* was the dominant species in wild birds in the Qinghai Lake, representing 55.6% (20/36) of the *Chlamydiaceae*-positive sample. As far as we know, there are no previous reports of *C. abortus* in birds in China. While *C. abortus* has been sporadically reported in Argentinian psittacines [34], its detection in Chinese wild birds constitutes a novel finding. To further characterize the *C. abortus*, phylogenetic analysis of *ompA* sequences (PQ303604–PQ303606) revealed that *C. abortus ompA* sequences were all closely related to their respective reference sequences available in GenBank and altogether showed a similar topology. 100% identity with *C. abortus* reference strain GN6 (CP021996.1) by nucleic acid comparison, originally isolated from aborted yak fetuses on the Qinghai-Tibetan Plateau [35]. This genomic congruence suggests potential cross-species transmission pathways: wild birds may act as reservoir hosts, contaminating shared aquatic environments through fecal shedding, thereby exposing sympatric yak populations to infection risks. Subsequent human infections could occur through direct livestock contact or aerosol exposure during pastoral activities. Such interspecies transmission dynamics could lead to wildlife population declines through reproductive impairments and ecosystem destabilization, necessitating comprehensive surveillance programs.

Our study also has some limitations. One is the modest sample size may limit the statistical power to fully assess the diversity and distribution of *Chlamydia* species in this ecosystem. Expanding the sample size in future studies would be valuable to confirm these initial insights and better understand transmission dynamics. Another is the exclusive use of randomly collected fecal samples introduces a potential overestimation risk, as multiple samples might originate from individual birds. Furthermore, the hypothesized cross-species transmission of *C. abortus* between wild birds and livestock, while supported by genetic identity, would be more conclusively demonstrated by concurrent testing of sympatric livestock in the same region. Future longitudinal studies should include such sampling to provide direct evidence of pathogen circulation across species boundaries. Finally, technical constraints affected molecular characterization: despite 36 *Chlamydia*-positive samples, successful amplification of 16 S rRNA, IGS-23 S rRNA, and *ompA* genes was only achieved in 5–6 cases due to low bacterial DNA loads. The reliance on qPCR without confirmatory sequencing for *C. psittaci* detection represents an additional constraint. Future investigations should incorporate multianatomic sampling (cloacal/oropharyngeal swabs) and meta-genomic approaches to enhance detection sensitivity and phylogenetic resolution.

## Conclusion

To summarize, this study illustrates that *C. abortus*, *C. avium*, and *C. psittaci* were detected in the wild birds in Qinghai Lake, China. *C. abortus* was the dominant species in wild bird in Qinghai Lake. Sequences of *C. abortus* obtained in this study were 100% identity with that of the *C. abortus* reference strain GN6 (CP021996.1). Furthermore, it may contribute to understanding the *Chlamydia* diversity and how it is transmitted.

## Supplementary Information

Supplementary Material 1. Figure. S1. Alignment of the two *ompA* sequences from *C. avium* reference strain (10DC88) and a field prevalent strain (QHL-WB-5) in the wild birds in China.

## Data Availability

All datasets generated for this study are included in the article.

## Abbreviations

AC: Avian chlamydiosis
RFLP: Restriction fragment length polymorphism
PBS: Phosphate-buffered saline
