# Supplementary figures and images for: Molecular prevalence of Chlamydia spp. in wild birds from Qinghai Lake, China
Source: Ir Vet J. 2025 Oct 30;78:28. doi: 10.1186/s13620-025-00314-2 (PMC12577048; doi:10.1186/s13620-025-00314-2)

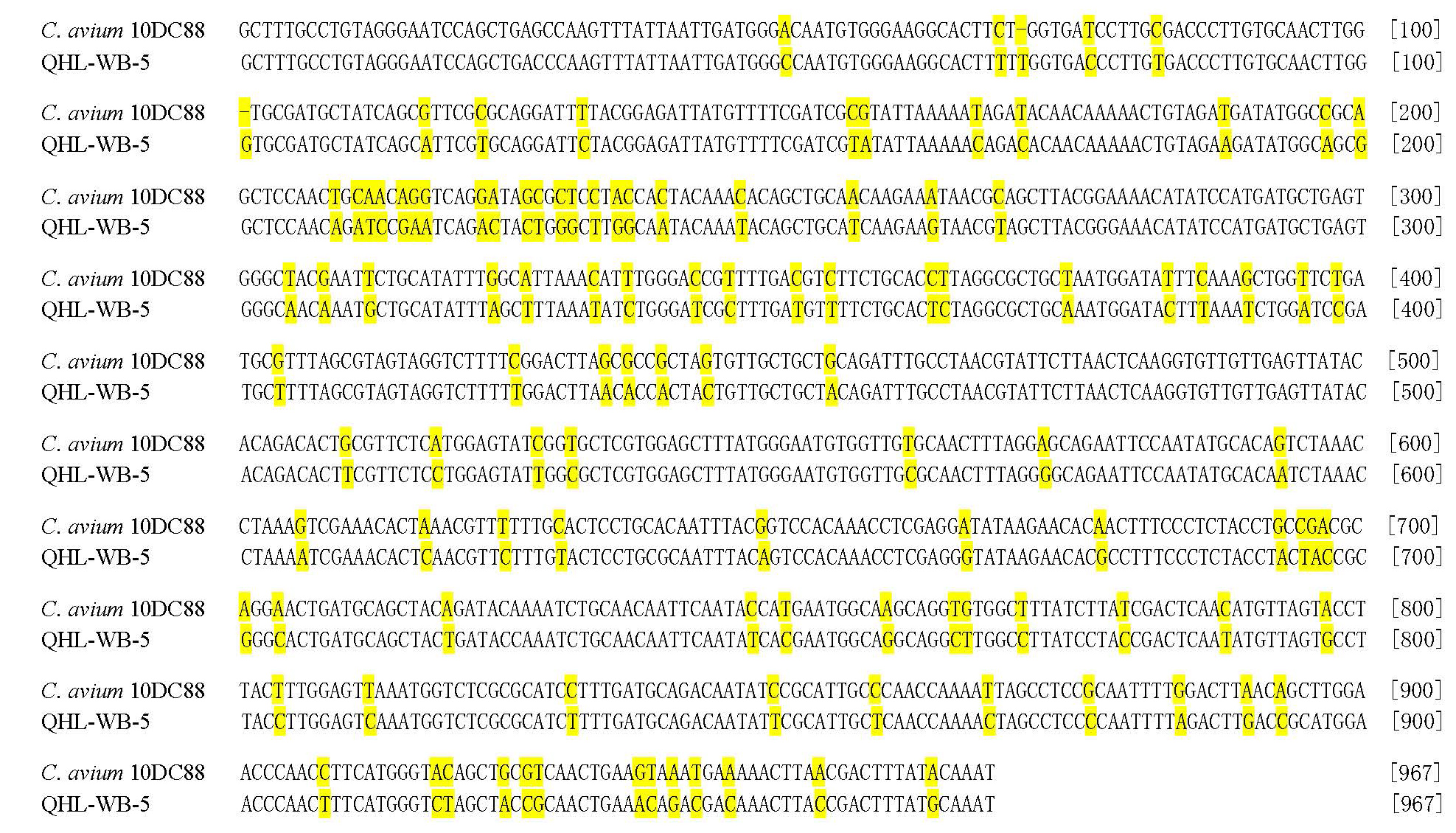

Supplement: Supplementary file 1 — Supplementary Material 1. Figure. S1. Alignment of the two ompA sequences from C. avium reference strain (10DC88) and a field prevalent strain (QHL-WB-5) in the wild birds in China. [file 13620_2025_314_MOESM1_ESM.jpg]
